# Alterations of the gut microbiota and short chain fatty acids in necrotizing enterocolitis and food protein-induced allergic protocolitis infants: A prospective cohort study

**DOI:** 10.3389/fcimb.2022.1030588

**Published:** 2022-11-21

**Authors:** Jing Xiong, Xing-Sheng Liao, Tong Yin, Xiao-Chen Liu, Lei Bao, Lu-Quan Li

**Affiliations:** ^1^ Neonatal Diagnosis and Treatment Center of Children’s Hospital of Chongqing Medical University, National Clinical Research Center for Child Health and Disorders, Ministry of Education Key Laboratory of Child Development and Disorders, International Science and Technology Cooperation Base of Child Development and Critical Disorders, Chongqing Key Laboratory of Pediatric, Chongqing, China; ^2^ Department of Neonatology, The first People’s Hospital of Jiulongpo District, Chongqing, China

**Keywords:** necrotizing enterocolitis, food protein-induced allergic protocolitis, gut mictobiota, short chain fatty acids, newborn

## Abstract

**Background:**

Even though presenting with similar clinical manifestations, necrotizing enterocolitis (NEC) and food protein-induced allergic protocolitis (FPIAP) have completely different treatments and prognosis. Our study aimed to quantify and evaluate differences in gut microbiota and short chain fatty acids (SCFAs) between infants with NEC and FPIAP to better identify these two diseases in clinical settings.

**Methods:**

A total of 43 infants with NEC or FPIAP in Children’s Hospital of Chongqing Medical University, China between December 2020 and December 2021 were enrolled. Stool samples were prospectively collected and froze. Infants defined as NEC were those who presented with clinical courses consistent with NEC and whose radiographs fulfilled criteria for Bell’s stage 2 or 3 NEC, while those who were healthy in appearance and had blood in the stool (visible or may be microscopic), had normal bowel sounds in physical examination, were resolved after eliminating the causative food, and/or had recurrence of symptoms after oral food challenge (OFC) were defined as FPIAP. Primers specific for bacterial 16S rRNA genes were used to amplify and pyrosequence fecal DNA from stool samples. Gas chromatography-mass spectrometry (GC-MS) technology was used to determine the concentrations of SCFAs.

**Results:**

Among the 43 infants, 22 were diagnosed with NEC and 21 were diagnosed with FPIAP. The microbial community structure in NEC infant stools differed significantly from those in FPIAP infant stools. NEC infants had significantly higher proportion of Actinobacteria and reduced proportion of Bacteroidetes compared with FPIAP infants, and the proportions of *Halomonas, Acinetobacter, Bifidobacterium*, and *Stenotrophomonas* in NEC infants were significantly higher than that of FPIAP infants. In addition, infants with NEC had significantly lower levels of acetic acid, propionic acid, butyric acid, isovaleric acid, and total SCFAs, and higher level of hexanoic acid as compared to the infants of the FPIAP group.

**Conclusions:**

The differences of gut microbiota composition and concentrations of SCFAs might represent suitable biomarker targets for early identification of NEC and FPIAP.

## Introduction

Necrotizing enterocolitis (NEC) is a destructive gastrointestinal disease that occurs primarily in preterm infants with high morbidity and mortality. It is associated with intestinal inflammation driven by microbiota and is characterized by an exaggerated inflammatory response and necrosis to the intestine resulting in the loss of intestinal barrier integrity and eventually multiple organ failure (Lu et al., 2014; Hackam and Caplan, 2018). A US-nationwide study reported that NEC affects about 8.9% of extremely preterm infants, among them 3.9% infants require surgery (Bell et al., 2022). According to a study by a UK specialist centre, the mortality for surgical NEC is 18.9% (Calvert et al., 2021). Common symptoms of NEC include gastric retention of enteral feedings, abdominal distension, and blood per rectum (Sowden et al., 2022). The onset of the disease is usually fulminant. Antibiotic therapy is usually used and therapeutic strategies for severe cases are limited and often useless.

Food protein-induced allergic proctocolitis (FPIAP) is a non-IgE-mediated gastrointestinal disorder with rising prevalence in food allergy. FPIAP is commonly caused by severe allergic reactions in the digestive system and has some overlapping clinical features with NEC, including hematochezia and diarrhea (Ohtsuka, 2015). The management of FPIAP relies upon avoidance of dietary triggers, with interval challenge to assess for resolution, which usually occurs in the first years of life (Feuille and Nowak-Węgrzyn, 2015). The exact prevalence of FPIAP is not well established. Study from North American revealed that the cumulative incidence of FPIAP was 17% over 3 years (Martin et al., 2020). Conversely, a large study of an Israeli birth cohort reported that the overall prevalence of FPIAP was low, at 0.16% (Elizur et al., 2012). Due to the lack of specific biomarkers, the diagnosis of FPIAP is mainly done by clinical history.

Intestinal dysbiosis has been proposed as one of the possible factors involved in the pathogenesis of NEC (Lindberg et al., 2020; Tarracchini et al., 2021; Thänert et al., 2021). Early life microbiota disruption had also been proven to be related to the development of metabolic disorders and allergies (Savage et al., 2018). Various studies reported that fecal microbiome from infants with NEC had increased relative abundances of Proteobacteria and Klebsiella and deceased relative abundances of Firmicutes and Bacteroidetes (Pammi et al., 2017a; Olm et al., 2019). While study described elevated relative abundances of Firmicutes and Bacteroidetes in FPIAP infants (Berni Canani et al., 2018a). Short chain fatty acids (SCFAs), mainly acetic acid, propionic acid and butyric acid, are the products of bacterial fermentation of carbohydrates in the intestines. Disruption in gut microbiota could subsequently cause the metabolic disorder of SCFAs.

NEC and FPIAP are two major diseases in preterm infants with overlapping clinical features but totally different treatment regimens. However, there is limited data in the literature comparing the gut microbiota and SCFAs between the NEC and FPIAP infants. Therefore, we firstly conducted this study to compare the gut microbiome and SCFAs of infants with NEC or FPIAP at a single tertiary center. We hypothesized that there would be differences in the gut microbial and SCFAs patterns between NEC and FPIAP infants. These differences might represent suitable biomarker targets for early identification of NEC and FPIAP.

## Materials and methods

### Standard protocol approval, registration, and patient consent

The study was approved by institutional review board for human studies of the Children’s Hospital of Chongqing Medical University (project approval No. 2020104) and registered at Chinese Clinical Trial Registry (Identifier: ChiCTR2000034672) (registration date, July 15, 2020). The study was designed to be prospective protocol, and informed consent was obtained from patient parents.

### Patient characteristics and sample collection

This prospective cohort trial was conducted in the tertiary referral NICU of Children’s Hospital of Chongqing Medical University between December 2020 and December 2021. A total of 168 neonates who presented clinical features of abdominal distension or hematochezia were recruited and the fecal samples of all the final included neonates were collected. All of the following neonates were excluded from the study: 1. neonates born with congenital intestinal disorders, 2. neonates who received probiotics before recruitment, 3. neonates whose parents refused the treatments, 4. neonates whose parents insist to discharge, 5. spontaneous intestinal perforation without radiographic evidence of NEC, and 6. neonates who lost the follow-up. In the end, 22 of the 168 recruited infants were diagnosed with NEC, while 21 of the 168 recruited infants were diagnosed with FPIAP.

Fecal samples of NEC or FPIAP patients were collected as soon as possible after the diagnosis was made within 24 hours. The samples were collected directly from the diapers by the nursing stuff using a sterile cotton swab, and then were placed into a sterile DNAase-, RNAase-, Eppendorf tube. All samples were frozen and stored at -80°C until processed.

### Case definition and clinical management

NEC was diagnosed based on the Vermont Oxford Network criteria (Vermont Oxford Network database) and staged according to Bell’s modified staging criteria (Kliegman and Walsh, 1987). The diagnosis of FPIAP is based on a careful and detailed history (including diet records), clinical manifestation of being healthy in appearance and being the presence of blood in the stool (visible or may be microscopic), physical examination of normal bowel sounds, remission of symptoms after eliminating the causative food, and/or recurrence of symptoms after oral food challenge (OFC) (Burks et al., 2012; Nowak-Węgrzyn et al., 2015; Meyer et al., 2020; Senocak et al., 2022). In addition, it is important to rule out other causes of blood in the stools in infancy such as anal fissures or infectious gastroenteritis (Thompson et al., 1996).

### Demographic and clinical variables

Neonatal and maternal medical record data were extracted from the medical record management system. Neonatal factors including: gestational age (GA), birth weight (BW), gender, age at sample collection, age at NEC/FPIAP diagnosis, antibiotic therapy before NEC/FPIAP diagnosis, and dietary information (type of feeding). Maternal factors including: mode of delivery, premature prolonged rupture of membranes (PPROM), meconium-staining amniotic fluid, intrauterine fetal distress, maternal hypertension, maternal diabetes, and use of antenatal corticosteroids.

### DNA extraction and 16S rRNA gene sequencing analysis

DNA of each sample was extracted using the E.Z.N.A.^®^ soil DNA Kit (Omega Bio-tek, Norcross, GA, USA) following the manufacturer’s instructions. The quality and concentration of DNA were determined by 1.0% agarose gel electrophoresis and a NanoDrop^®^ ND-2000 spectrophotometer (Thermo Scientific Inc., USA) and kept at -80 °C prior to further use. The V3-V4 region of 16S rRNA gene was amplified using primers 338F (5′-ACTCCTACGGGAGGCAGCA-3′) and 806R (5′-GGACTACHVGGGTWTCTAAT-3′) by an ABI GeneAmp^®^ 9700 PCR thermocycler (ABI, CA, USA). For each extracted DNA sample, The PCR reaction mixture contains 4 μL 5 × Fast Pfu buffer, 2 μL 2.5 mM dNTPs, 0.8 μL each primer (5 μM), 0.4 μL Fast Pfu polymerase, 10 ng of template DNA, and ddH2O to a final volume of 20 µL. PCR was performed with the following conditions: initial denaturation at 95 °C for 3 min, followed by 27 cycles of denaturing at 95 °C for 30 s, annealing at 55 °C for 30 s and extension at 72 °C for 45 s, and single extension at 72 °C for 10 min, and end at 4 °C. All samples were amplified in triplicate.

### PCR products purification

The amplification products were separated by 2% agarose gel electrophoresis, purified using the AxyPrep DNA Gel Extraction Kit (Axygen Biosciences, Union City, CA, USA) according to manufacturer’s instructions, and quantified with a Quantus™ Fluorometer (Promega, USA).

### Library preparation and sequencing

DNA library preparation was performed using the TruSeq DNA PCR-Free Sample Preparation Kit (Illumina, San Diego, CA), following the manufacturer’s instructions. Sequencing was performed on Novaseq6000 instrument (Illumina, San Diego, CA), following the manufacturer’s instructions.

### Bioinformatic analysis

Bioinformatic analysis of the gut microbiota was carried out using the Majorbio Cloud platform (https://cloud.majorbio.com). Sequences were divided into operational taxonomic units (OTUs) using similarity levels with a cutoff of 97% similar. Bacterial OTU representative sequences were assigned to a taxonomic lineage by a Ribosomal Database Project (RDP) classifier version 2.2 against the 16S rRNA gene database (Silva v138). Based on the OTUs information, rarefaction curves and α-diversity indices including Shannon index, Simpson index, Ace index, and Chao1 index were calculated with Mothur v1.30.1 (Schloss et al., 2009). The similarity among the microbial communities in different samples was determined by principal coordinate analysis (PCoA) based on Bray-curtis dissimilarity using Vegan v2.5-3 package. The linear discriminant analysis (LDA) effect size (LEfSe) (http://huttenhower.sph.harvard.edu/LEfSe) was performed to identify the significantly abundant taxa (phylum to genera) of bacteria among the different groups (LDA score≥4, *P* < 0.05) (Segata et al., 2011). Correlation heatmap was conducted to explore the relationship between SCFAs concentrations and the gut microbiota composition based on *Spearman rank correlation* in R.

### SCFAs analysis

Fecal SCFAs concentration was determined by using gas chromatography-mass spectrometry (GC-MS) technology (Termo TRACE 1310-ISQ LT, America) as follows: Briefy, fecal pellets were ground twice for three minutes, placed in an ice bath for 30 minutes, held at 4°C for 30 minutes, and centrifuged at 13,000 rpm, at 4 °C for 15 minutes. In addition, ethyl acetate was added to SCFAs (including acetic, propionic, butyric, isovaleric, hexanoic acid, and the total SCFAs) and 2-ethylbutyric acid to obtain standard concentration gradients. Then, a small sample of the supernatant (1 μL) and the standard solution were injected into the column and used for detection by GC-MS. Lastly, Masshunter quantitative software (version10.0.707.0; Palo Alto, USA) was used to automatically identify and integrate target SCFAs. The SCFAs concentrations of each sample were calculated based on standard curves.

### Statistical analysis

All data were analyzed using SPSS version 24.0 software (SPSS Inc., USA). Data exhibiting a normal distribution were described as the mean with standard deviation (SD) and were analyzed by means of Student’s t-test or one-way analysis of variance (ANOVA). Non-normally distributed measurement data were presented as the median (interquartile range) and were analyzed by means of the Wilcoxon rank-sum test. Categorical data were compared using chi-square tests or Fisher’s exact test, when appropriate. Receiver operating characteristic (ROC) curves and all figures were generated with GraphPad Prism (version 9.0; California, USA). Two sided *P* values < 0.05 were considered statistically significant.

## Results

### Subjects

**Figure 1:**
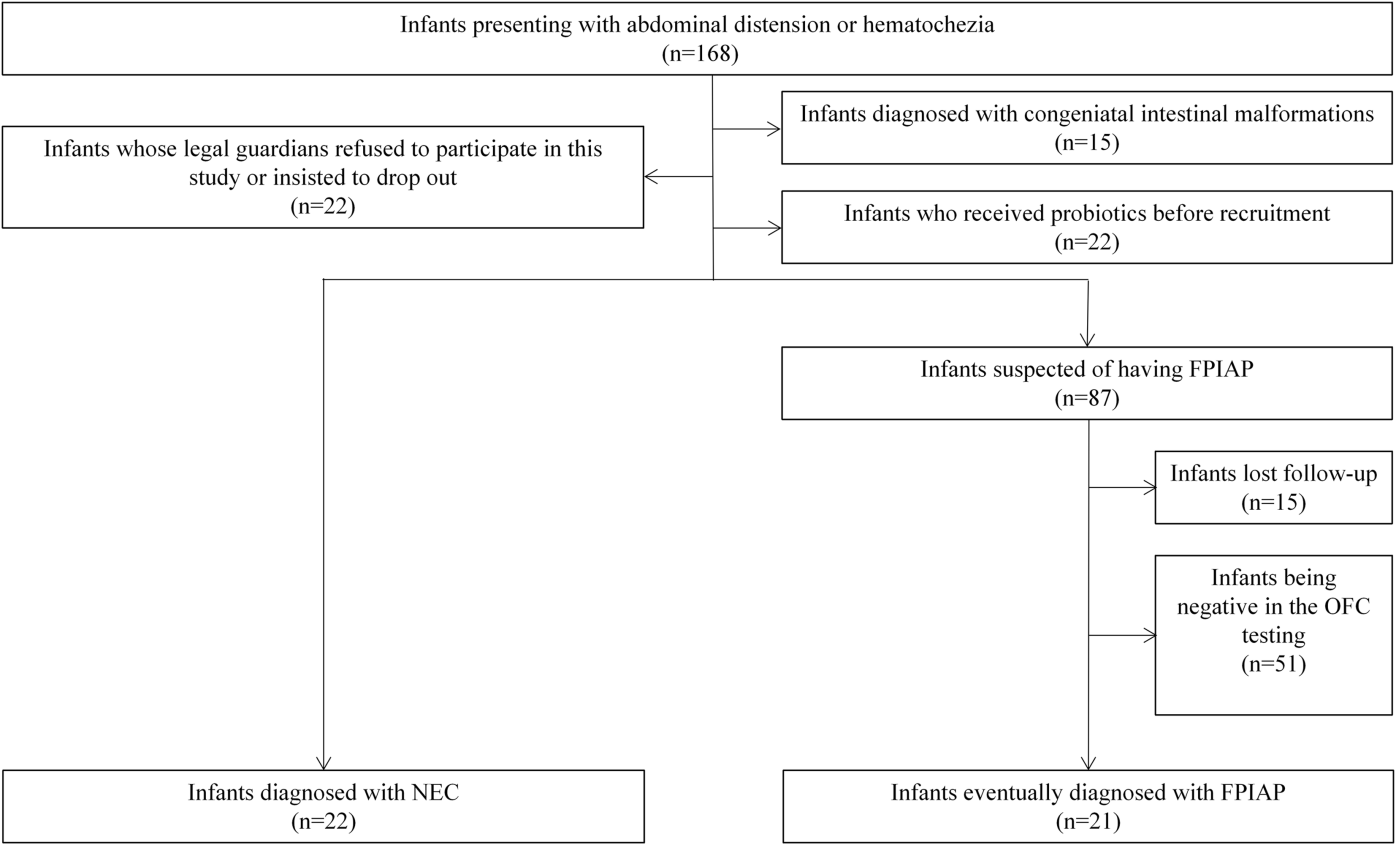
Flow diagram of the inclusion and exclusion processes of the study.

A total of 168 infants presenting abdominal distension or hematochezia over one year were enrolled prospectively. 22 (13.1%) of 168 infants developed NEC, while 21 (12.5%) of these infants developed FPIAP (**Figure 1**). The basic clinical characteristics are shown in **Table 1**. The median age of onset of NEC was 11.6 days (interquartile range [IQR], 6.8-16.0 days), while that of FPIAP was 15.2 days (IQR, 11.0-22.0 days). In NEC infants, 9.1% of them were exclusively breast-fed, 72.7% were fed with cow’s milk (CM)-based formula, and 18.2% were fed by both breast milk and CM-based formula before the onset of symptoms. While the percentages of these three feeding patterns in FPIAP infants were 19.1%, 61.9%, and 19.0%, respectively.

**Table 1:** Baseline characteristics of the newborns between the NEC and FPIAP groups.

	NEC (n=22)	FPIAP (n=21)	*P* value
Gestational age (weeks)	35.5 ± 2.2	36.5 ± 1.4	0.105
Birth weight (g)	2542.9 ± 699.9	2715 ± 482.3	0.355
Male, n (%)	17 (77.3)	14 (66.7)	0.438
Age at sample collection (days)	11.6 (6.8-16.0)	15.2 (11.0-22.0)	0.114
Age at NEC/FPIAP diagnosis (days)	11.6 (6.8-16.0)	15.2 (11.0-22.0)	0.114
Rupture of membranes (>18h), n (%)	8 (36.4)	4 (19.0)	0.206
Meconium-staining amniotic fluid, n (%)	2 (9.1)	1 (4.8)	0.578
Intrauterine fetal distress, n (%)	4 (18.2)	2 (9.5)	0.413
Maternal hypertension, n (%)	3 (13.6)	0 (0)	0.079
Maternal diabetes, n (%)	5 (22.7)	2 (9.5)	0.241
Prenatal use of corticosteroids, n (%)	7 (31.8)	2 (9.5)	0.072
Vaginal birth, n (%)	17 (77.3)	14 (66.7)	0.438
Feeding pattern before the onset of symptoms			
Breast feeding, n (%)	2 (9.1)	4 (19.1)	0.412
CM-based formula, n (%)	16 (72.7)	13 (61.9)	0.526
Mixed feeding, n (%)	4 (18.2)	4 (19.0)	1.000
Antibiotic therapy before NEC/FPIAP diagnosis, n (%)	9 (40.9)	3 (14.3)	0.052

Data are mean (SD), median (IQR), or n (%), unless otherwise specified. CM, cow’s milk; NEC, necrotizing enterocolitis; FPIAP, food protein-induced allergic proctocolitis.

### Characterization of gut microbiota

DNA sequences from the 43 fecal samples were analyzed, with a total of 4,438,641 sequences and an average length of 465 base pairs (bps). The rarefaction curves based on sobs index, the Shannone curves based on Shannon index, and the species accumulation curves of each group demonstrated that the sequencing data and depth, and the sample size were sufficient (**Supplementary Figure S1**).

**Figure 2:**
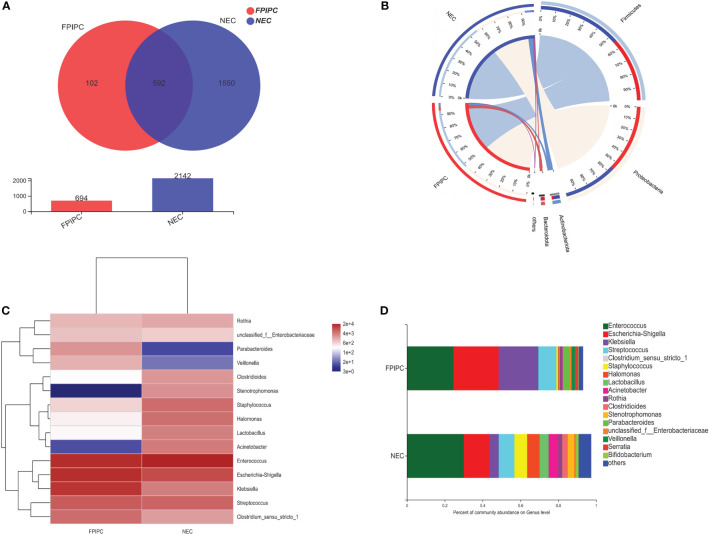
Shared and unique microbiota between the necrotizing enterocolitis (NEC) and food protein-induced allergic protocolitis (FPIAP) groups. **(A)** The Circos plot shows each main phyla between the NEC and FPIAP groups. Outer bars show the percentage of reads in a category that are connected to the category at the other end of the drawn band. **(B)** The heatmap displays genus-level changes (rows) between the samples of NEC and FPIAP groups (columns). The variation of each genus is indicated by a gradient of color from blue (decease) to red (increase). **(C)** Genus-level taxonomic composition of the NEC and FPIAP infants. Relative abundances are reported on the horizontal axis and the two groups on the vertical axis **(D)**.

A Venn diagram was used to indicate the differences of bacterial populations between the two groups. The Venn diagram revealed that 592 OTUs were shared between the NEC and FPIAP groups, while 1550 and 102 OTUs were unique to NEC infants and FPIAP infants, respectively (**Figure 2A**). Suggesting that the richness of gut microbiota in NEC infants was higher than that of the FPIAP infants, and there were shared or specific gut microbiota between the two groups. As shown in the circos plot, Firmicutes, Proteobacteria, Actinobacteriota, and Bacteroidota were dominant in both NEC and FPIAP infants at the phylum level (**Figure 2B**). The community abundance on genus level of NEC and FPIAP infants was shown in **Figures 2C, D**, which showed noticeable discrepancies in community structure between infants with NEC and FPIAP (**Figures 2C, D**).

### Microbial diversity

**Figure 3:**
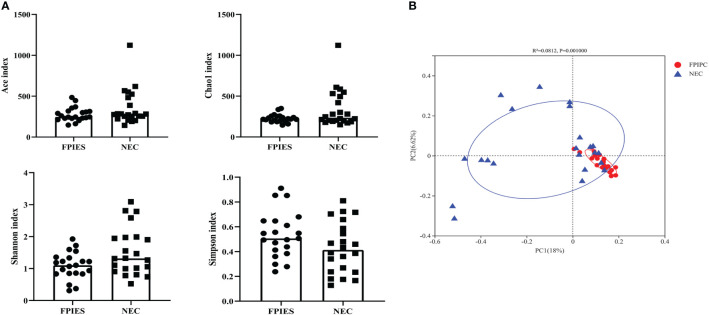
Alpha and beta diversity between the necrotizing enterocolitis (NEC) and food protein-induced allergic protocolitis (FPIAP) groups. There were no significant differences between the NEC and FPIAP groups in the ace index, Chao1 index, Shannon index, and Simpson index (*P*>0.05). **(A)** There was a significant difference in beta diversity between the NEC and FPIAP groups (*P*<;0.05). **(B)** *N*=22 for NEC and *N*=21 for FPIAP.

**Figure 4:**
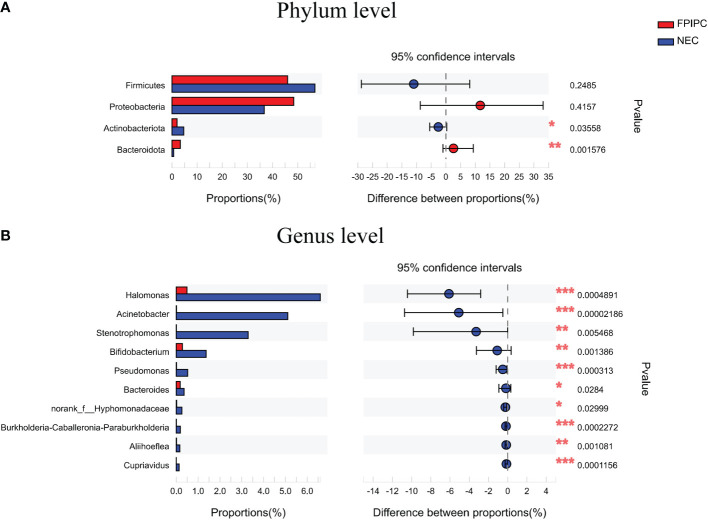
Relative abundances at the phylum level **(A)** and the genus level **(B)** in the necrotizing enterocolitis (NEC) and food protein-induced allergic protocolitis (FPIAP) groups. There were significant differences between the NEC and FPIAP groups in gut microbiota at the phylum and genus levels. *P<0.05, **P<0.01, ***, and *P*<0.001. Data was presented as means±SEM, *N*=22 for NEC and *N*=21 for FPIAP.

The α-phylogenetic diversity indexes were analyzed to explore the community richness and diversity in two groups. No significant differences were observed between the two groups in terms of Shannon, Simpson, Ace, and Chao1 indexes (**Figure 3A**). The overall microbial structure was then analyzed in each group at the phylum and genus levels. The results showed that Firmicutes, Proteobacteria, Bacteroidota, and Actinobacteriota were the most abundant bacteria in the two groups and constituted over 90% of the total bacteria at the phylum level. Infants in the NEC group had significantly higher proportion of Actinobacteriota and reduced proportion of Bacteroidota compared with infants in the FPIAP group (*P*<;0.05, **Figure 4A**). While no significant difference was observed between the two groups in terms of Firmicutes and Proteobacteria. At the genus level, infants in the NEC group had significantly higher proportions of *Halomonas, Acinetobacter, Bifidobacterium*, and *Stenotrophomonas* as compared to the infants of the FPIAP group (*P*<;0.05, **Figure 4B**).

Given these findings, principle coordinate analysis (PCoA) of unweighted UniFrac distances was used to estimate β-diversity of gut microbiota between the two groups. The NEC group had more variability compared with the FPIAP group. PCoA of unweighted UniFrac distance (quantitative, R^2^ = 0.0812, *P* = 0.001) showed that the samples in the FPIAP group were ordinated closely, while the samples in the NEC group were separated obviously, indicating differences in bacterial structure in the NEC group (**Figure 3B**).

**Figure 5:**
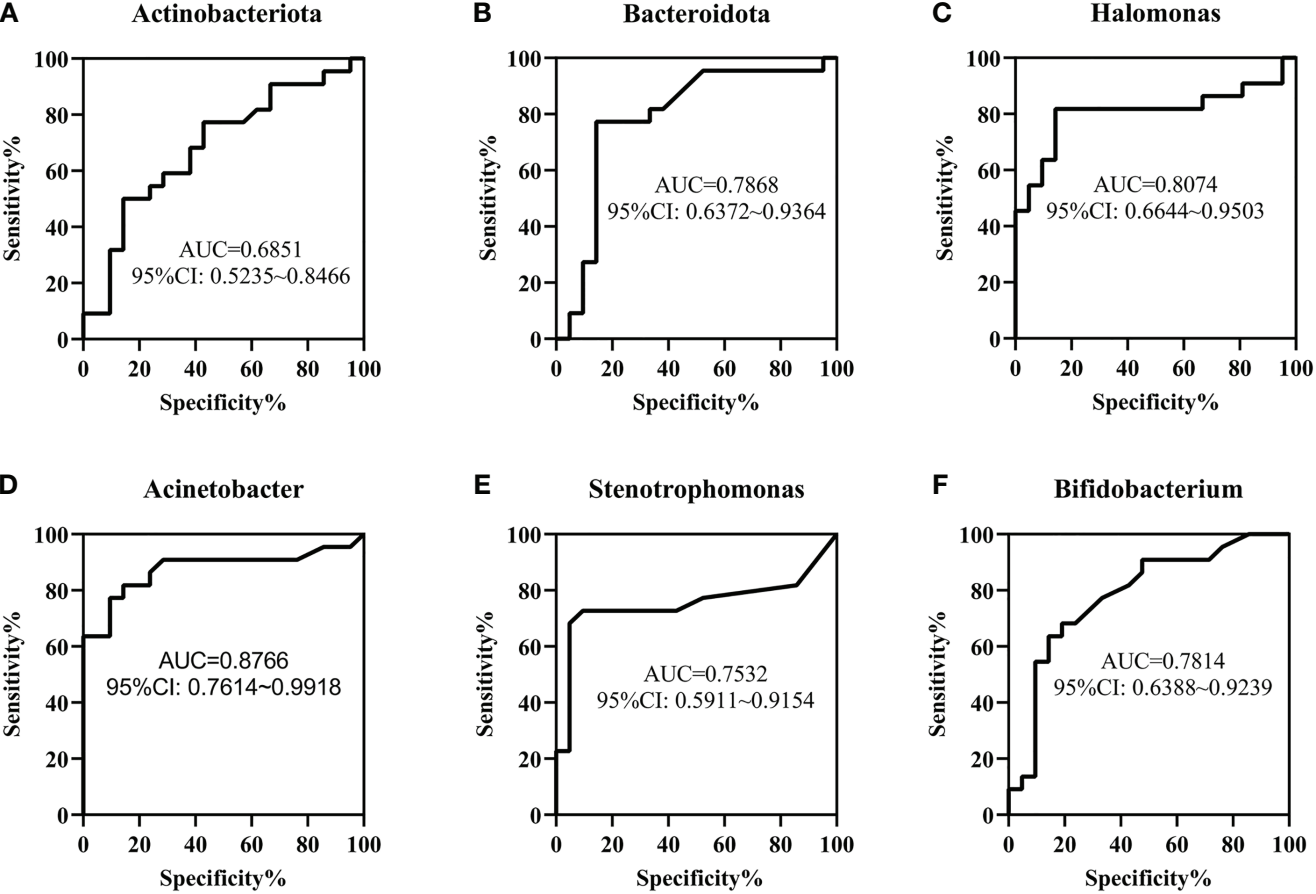
The value of gut microbiota in the early identification of necrotizing enterocolitis (NEC) and food protein-induced allergic protocolitis (FPIAP) by receiver operating characteristic (ROC) analysis. The area under curves (*AUCs)* of Actinobacteriota **(A)**, Bacteroidota **(B)**, *Halomonas* **(C) **, *Acinetobacter ***(D)**, *Stenotrophomonas ***(E) **, *and Bifidobacterium* **(F)** were 0.6851, 0.7868, 0.8074, 0.8766, 0.7532, and 0.7814, respectively. *N*=22 for NEC and *N*=21 for FPIAP.

To determine the value of gut microbiota in identifying FPIAP and NEC in the early stage, ROC curves of Actinobacteriota, Bacteroidota, *Halomonas, Acinetobacter, Stenotrophomonas, and Bifidobacterium* were performed, and the area under curves (*AUCs)* were 0.6851, 0.7868, 0.8074, 0.8766, 0.7532, and 0.7814, respectively (**Figure 5**).

### LEfSe analysis

**Figure 6:**
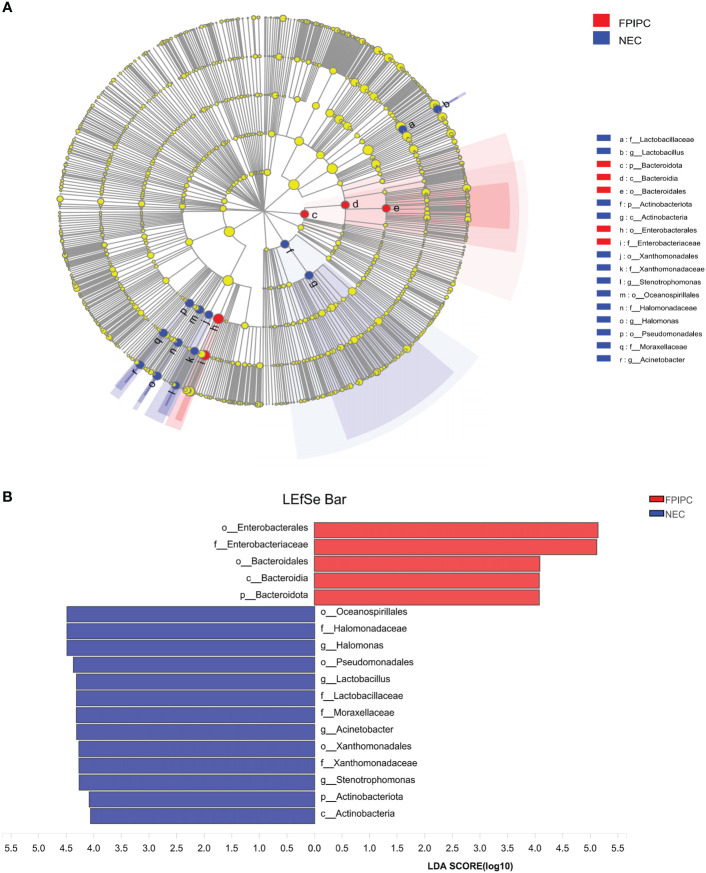
Differentially abundant taxa between the necrotizing enterocolitis (NEC) and food protein-induced allergic protocolitis (FPIAP) groups analyzed by Linear discriminant analysis (LDA) effect size (LEfSe) were shown in cladogram and histogram. **(A)** Comparative analysis of the gut microbiota by LEfSe: the cladogram shows bacterial taxa significantly higher in the group of infants of the same color, in the gut microbiota between NEC and FPIAP infants; **(B)** Gut microbiota analysis *via* LDA score between NEC and FPIAP infants. *N*=22 for NEC and *N*=21 for FPIAP.

Differential abundant phylotypes between the two groups were further evaluated by LEfSe using linear discriminant analysis (LDA) (LDA score≥4). This threshold guarantees that the meaningful taxa is compared and eliminates most of rare taxa. The figure generated in the LEfSe analysis (**Figure 6A**) shows the taxonomic groups with the largest differences between the two groups at various levels. The histogram (**Figure 6B**) shows the differences in 18 phylotypes between the two groups. At the family level, the abundance of Enterobacteriaceae in the fecal microbiota was higher in the FPIAP group, whereas the abundance of *Halomonadaceae, Lactobacillaceae, Moraxellaxceae*, and *Xanthomonadaceae* was higher in the NEC group. There were four genus levels (*Halomonas, Lactobacillus, Acinetobacter*, and *Stenotrophomonas*) differences between the two groups, and the abundance of these four genus was higher in the NEC group as compared to the FPIAP group.

### SCFAs production in NEC and FPIAP infants

**Figure 7:**
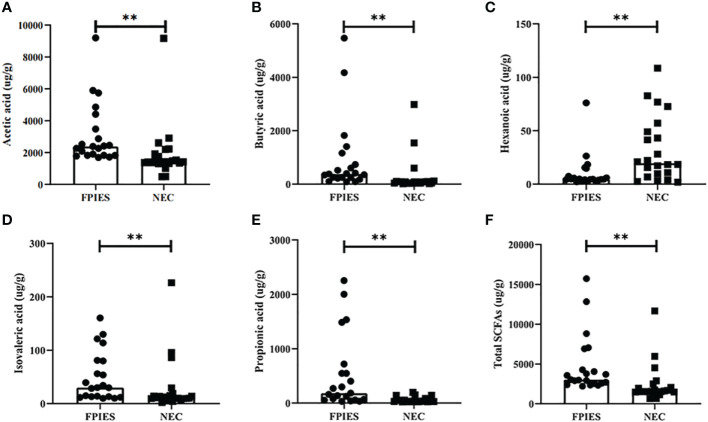
Gas chromatography-mass spectrometry (GC-MS) analysis of short chain fatty acids (SCFAs) in fecal samples from necrotizing enterocolitis (NEC) infants and food protein-induced allergic protocolitis (FPIAP). **(A)** acetic acid, **(B)** butyric acid, **(C)** hexanoic acid, **(D)** isovaleric acid, **(E)** propionic acid, and **(F)** Total SCFAs. **P<;0.01. Data was presented as means±SEM, *N*=22 for NEC and *N*=21 for FPIAP.

In this study, GC-MS was used to investigate the concentrations of SCFAs in each sample. The results revealed that compared to the infants with FPIAP, infants with NEC had significantly lower levels of acetic acid, propionic acid, butyric acid, isovaleric acid, and total SCFAs, and higher level of hexanoic acid (all *P*<;0.05) (**Figure 7**).

**Figure 8:**
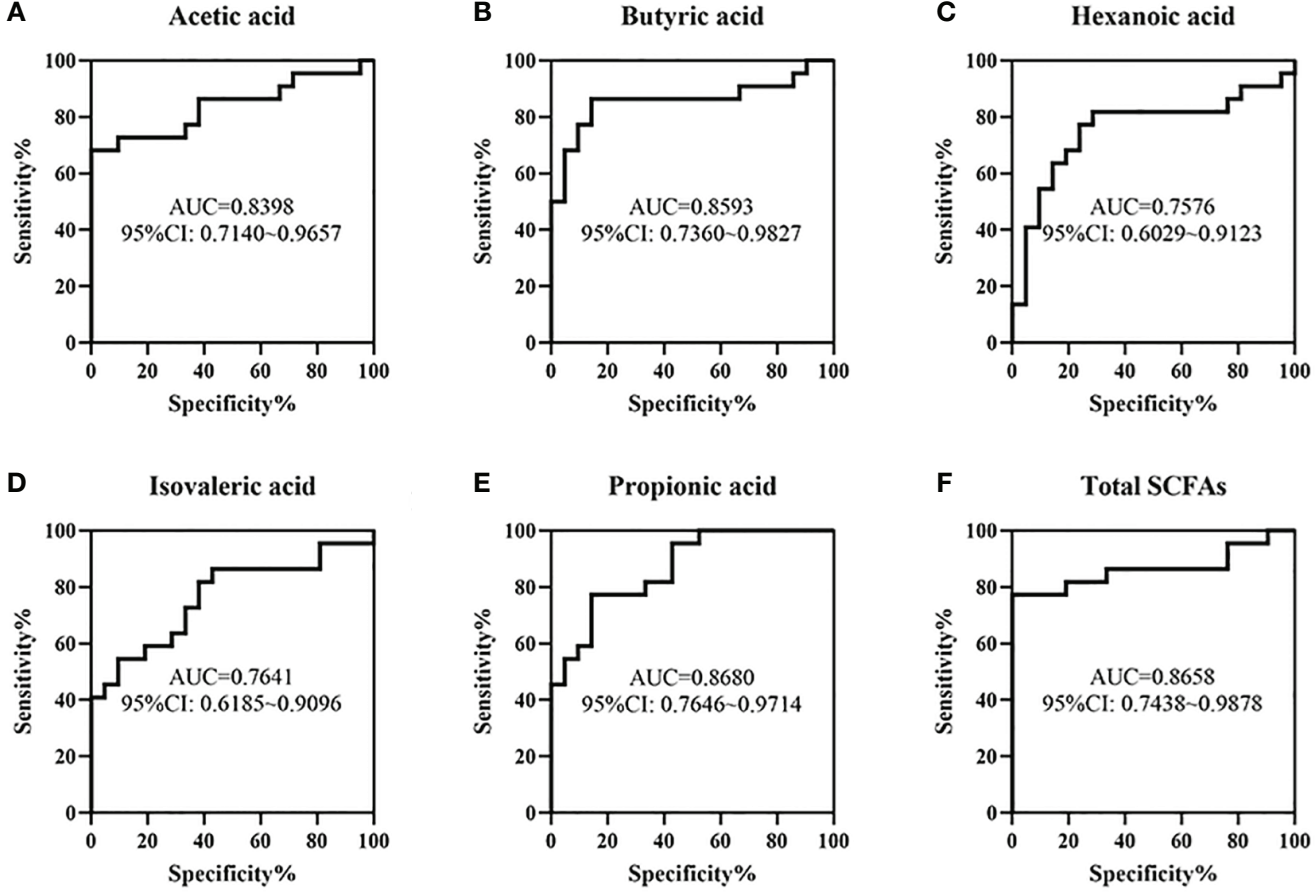
The value of some short chain fatty acids (SCFAs) in the early identification of necrotizing enterocolitis (NEC) and food protein-induced allergic protocolitis (FPIAP) by receiver operating characteristic (ROC) analysis. The area under curves (*AUCs)* of acetic acid **(A)**, butyric acid **(B)**, hexanoic acid **(C)**, isovaleric acid **(D)**, propionic acid **(E)**, and Total SCFAs **(F)** were 0.8398, 0.8593, 0.7576, 0.7641, 0.8680, and 0.8658, respectively. *N*=22 for NEC and *N*=21 for FPIAP.

ROC curves fore these metabolites were conducted to evaluate the value of SCFAs in the early identification of NEC and FPIAP. The results showed that the *AUCs* of acetic acid, butyric acid, hexanoic acid, isovaleric acid, propionic acid, and total SCFAs were 0.8398, 0.8593, 0.7576, 0.7641, 0.8680, and 0.8658, respectively (**Figure 8**).

### Relationship between SCFAs and the gut microbiota

**Figure 9:**
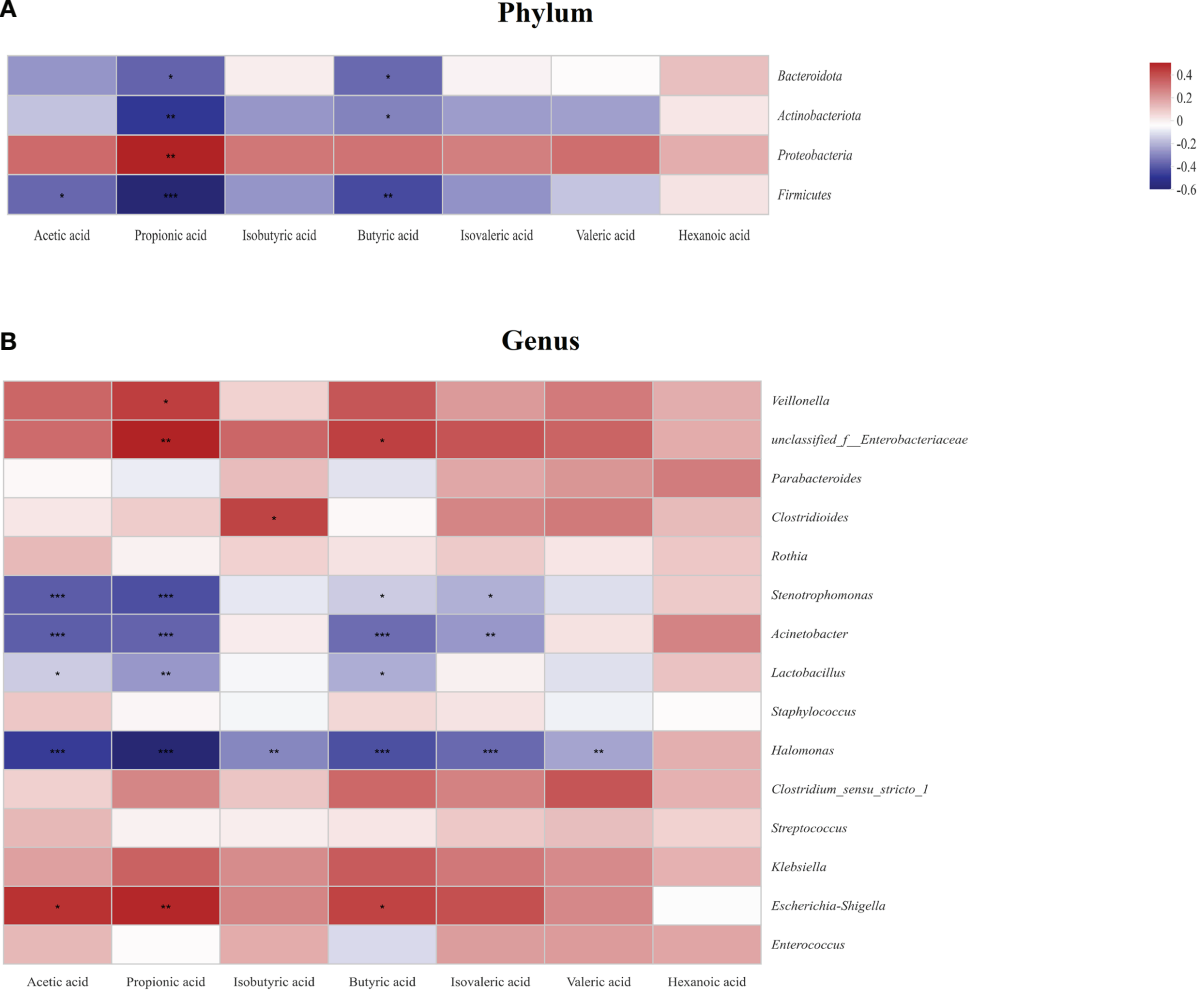
Relationship between the gut microbiota and short chain fatty acids (SCFAs) at the phylum level **(A)** and the genus level **(B)** in the study. Some flora and SCFAs were negatively related, represented in blue, and others were positively related, represented in red. The darker the color, the higher the correlation was. *P<0.05, **P<0.01, and ***P<0.001. Data was presented as means±SEM, N=22 for necrotizing enterocolitis (NEC) and N=21 for food protein-induced allergic protocolitis (FPIAP).

To explore the relationship between SCFAs and the gut microbiota, a heatmap was conducted as shown in **Figures 9A, B**. At the phylum level, propionic acid and butyric acid were all negatively correlated with Bacteroidota, Actinobacteriota, Proteobacteria, and Firmicutes, and acetic acid was negatively correlated with Firmucutes. In the contrary, propionic acid was positively correlated with Proteobacteria (*P*<;0.05) (**Figure 9A**). At the genus level, most SCFAs were negatively correlated with *Stenotrophomonas*, *Acinetobacter*, *Lactobacillus*, and *Halomonas*. In the contrary, acetic acid, propionic acid, and butyric acid were positively correlated with *Escherichia-Shigella*, and isobutyric acid was positively correlated with *Clostridioides* (*P*<;0.05) (**Figure 9B**).

## Discussion

New biomarkers for the early identification of FPIAP and NEC are important. In this study, high throughput 16S rRNA gene sequencing and GC-MS techniques were used to compare the gut microbial profiles and diversity and metabolite characteristics in infants with NEC and FPIAP. The results confirmed that there are significant differences between the NEC and FPIAP groups in the main component of the gut microbiota, with differences between the two groups in species composition at different classification levels. In addition, significant differences were also observed between the two groups in terms of the fecal SCFAs concentrations, including acetic acid, propionic acid, butyric acid, isovaleric acid, hexanoic acid, and total SCFAs. These findings could provide value for the early identification of FPIAP and NEC in clinical settings.

At the phylum level, our study found that Firmicutes and Proteobacteria constituted the main component of intestinal microbiome in both NEC and FPIAP infants. As compared to the infants with FPIAP, infants in the NEC group had a remarkably lower number of Bacteroidota and higher number of Actinobacteriota. Previous studies have reported that the gut characteristic bacterial populations in healthy newborns are dominant in *Lactobacillus* and *Bifidobacterium* in term neonates, and *Enterobacteriaceae*, *Veillonella*, *Enterococcus*, and *Staphylococcus* in preterm neonates, respectively (Tirone et al., 2019), which are different from those in NEC or FPIAP infants of our study. There are four main phyla in the gut microbiota, including Firmicutes, Bacteroidota, Proteobacteria, and Actinobacteriota (Faith et al., 2013). The two dominant phyla, Firmicutes and Bacteroidota, represent over 90% of the total gut microbiota community (Magne et al., 2020), while members of Proteobacteria and Actinobacteriota are less abundant. Bacteroidota contain a large repertoire of genes involved in acquisition and metabolism of polysaccharides (Mahowald et al., 2009), and *Bacteroides* species are reported to be able to alter gut permeability (Curtis et al., 2014; Hua et al., 2016). Bacteroidota species have either anti-inflammatory effects or are involved in the process of proteolysis, however, some Bacteroidota species are pathogetic. The previous studies demonstrated that low level of Bacteroidota is associated with allergic disease and factors related to allergic disease, such as a Western lifestyle and cesarean section delivery (De Filippo et al., 2010; Abrahamsson et al., 2012). In contract, an enrichment of *Bacteroides* was found in non-IgE-mediated Cow’s milk allergy children (Berni Canani et al., 2018b). Specifically, Kirjavainen et al. found high abundance of *Bacteroides* in the gut microbiome of infants with a high degree of milk allergy, early onset atopic eczema, and a strong family history of atopic disorders (Kirjavainen et al., 2002). A meta-analysis by Pammi et al. reported that fecal microbiome from infants with NEC had increased relative abundances of Proteobacteria and decreased relative abundances of Firmicutes and Bacteroidota (Pammi et al., 2017b), consistent with our results. In our study, the abundance of Actinobacteriota was higher in the NEC group as compared to the FPIAP group, which consistent with a previous study by Torrazza et al., in which a higher proportion of Actinobacteria was observed in NEC cases compared to controls (Torrazza et al., 2013). Actinobacteriota are Gram-negative bacteria with linear colonies and numerous species, such as *Bifidobacterium*, which are involved in immune modulation and metabolic activities. These findings indicated that dysbiosis of the gut microbiota plays an important role in the development of NEC and FPIAP.

At the genus level, our study also showed that differences in the gut microbiota at the genus level were notable between the NEC and FPIAP groups. The abundances of several genera of *Halomonas, Acinetobacter, Bifidobacterium*, and *Stenotrophomonas* were remarkably higher in the NEC group as compared to the FPIAP group. Belongs to the class Gammaproteobacteria and the family Halomonadaceae, *Halomonas* is a Gram-negative, aerobic, rod-shaped, extremely halotolerant bacteria, which has been found to have cytotoxic activity (Kim et al., 2013; Cheffi et al., 2021). *Stenotrophomonas* is an opportunistic pathogen of significant concern to susceptible patient populations, it can cause various nosocomial and community-acquired infections in humans and shows low susceptibility to many antibiotics (Sánchez, 2015; Brooke, 2021). To date, no studies have been carried out to explore the correlation between *Stenotrophomonas* and NEC or FPIPC infants. *Acinetobacter* is a gram-negative bacterum and is one of the most significant emerging multidrug-resistant pathogens. It is the cause of various hospital-acquired diseases including septicemia, pneumonia, and wound infections (Geisinger et al., 2019). The genus *Bifidobacterium* is included within the phylum Actinobacteria and plays an important role in digestion and gut immunity. Previous studies have shown that some *Bifidobacterium* species have proteolytic activity (De Palma et al., 2010; de Almeida et al., 2020), which helps the protein absorption. Chen et al. found that the abundance of *Bifidobacterium* increased significantly in children with food protein allergy (Chen et al., 2016). Some studies have found that children received *Bifidobacterium* supplementation had significantly reduced allergic symptoms (Ismail et al., 2016; Liu et al., 2018). Decrease in the abundance of *Bifidobacterium* leads to a decrease in protein absorption and transformation, which may be the cause of FPIAP. Generally, the significant differences in abundance of *Halomonas*, *Acinetobacter*, *Bifidobacterium*, and *Stenotrophomonas* between the two groups may serve as biomarkers for NEC and FPIAP infants and suggest a role for the gut microbiota in the pathogenesis of the main symptoms of the disorder.

In this study, no significant differences were observed in mode of delivery, feeding patterns, and antibiotic exposure between the NEC and FPIAP groups, suggesting that the microbial diversity between the two groups in the present study may be associated with other factors. Substantial evidence suggested that the abundance and diversity of microbiota could be affected by multiple factors, including mode of delivery, feeding patterns (i.e., breast milk, formula, or both), and antibiotic exposure. Study reported that the microbial diversity was lower in infants delivered *via* C-section than in infants delivered vaginally (Korpela et al., 2018; Korpela et al., 2018; Lundgren et al., 2018). In fecal samples of infants born by vaginal delivery, the *Bifidobacterium* genus was predominant (Biasucci et al., 2008), followed by *Bacteroides* and *enterobacteria* (Fallani et al., 2010). In addition, Willers et al. showed that infants born by vaginal delivery had higher levels of S100 proteins as compared to infants born by cesarean delivery, which was associated with higher abundance of Actinobacteria and *Bifidobacteriaceae*, and lower abundance of Gammaproteobacteria-particularly opportunistic *Enterobacteriacea* (Willers et al., 2020). Investigating to the role of diet, Pammi et al. reported that significant differences in microbial diversity were observed among infants with different feeding types (Pammi et al., 2017a). In particular, formula-fed infants who developed NEC had more Proteobacteria and less Firmicutes compared to breast milk-fed controls (Pammi et al., 2017a). Investigating to antibiotic exposure, study also found that OTU richness between control infants who didn’t received antibiotics and NEC infants who received antibiotics differed significantly (Pammi et al., 2017a). Antibiotic treatment decreases α-diversity of the individual’s microbiome (Yassour et al., 2016). Besides, maternal treatment with antibiotics prior to delivery has also been related to a decrease in microbial diversity in infants, especially lacking *Bifidobacterium*, which is a genus regarded as favorable (Aloisio et al., 2014). The possible reason for the inconsistence between our findings and the above studies is due to the small sample size, and studies with a larger sample size are needed.

In the current study, NEC infants had significantly lower levels of acetic acid, propionic acid, butyric acid, isovaleric acid, and total SCFAs, and higher level of hexanoic acid compared with the FPIAP infants. SCFAs, which are produced by fermentation of dietary fibre by gut microbiota, are potential mediators involved in the intestinal immune function, including the inhibition of the production of pro-inflammatory factors and the maintenance of gut barrier function (Tedelind et al., 2007). Among the most common SCFAs, acetic acid, propionic acid, and butyric acid account for 90-95% of SCFAs in the human colon (Soldavini and Kaunitz, 2013). Some studies reported that acetic acid, propionic acid, and butyric acid can be produced by the fermentation of *Ruminococcus* (Lin et al., 2021; Liang et al., 2021). Besides, Some *Lactobacillus* strains, including *L. rhamnosus* GG, *L. gasseri* PA 16/8, *L. salivarius spp salcinius* JCM 1230, *L. agilis* JCM 1048, and *L. acidophilus* CRL 1014 were reported to participate in the production of acetic acid, propionic acid, and butyric acid (Markowiak-Kopeć and Śliżewska, 2020). Acetic acid is the most abundant SCFAs in the colon and constitutes over half of the total SCFAs content in the feces (Ziętek et al., 2021). Propionic acid is produced primarily by Bacteroidetes and Firmicutes (Russell et al., 2013). Acetic, propionic, and butyric acid have been shown to induce apoptosis (Kotunia et al., 2010), and butyric acid has been shown to exert the most significant anti-inflammatory property of all SCFAs and can improve the intestinal barrier function and mucosal immunity (Liu et al., 2018). Isovaleric acid and hexanoic acid are putrefactive acids generated by the unabsorbed amino acids or proteins reaching the intestines. Adequate balance of microbiota and metabolites could allow intestinal homeostasis and immunologic tolerance to food antigens, while imbalance of gut microbiota and their metabolites (SCFAs) possible influence key immunologic events that enhance allergic sensitization to food antigens. Therefore, the differences of the concentrations of SCFAs and the components of gut microbiota between the NEC and FPIAP groups could provide new strategies for the differential diagnosis of NEC and FPIPC.

In addition, we explored the value of the gut microbiota and SCFAs in the early identification of NEC and FPIAP and the results showed that the *AUCs* of Actinobacteriota, Bacteroidota, *Halomonas, Acinetobacter, Stenotrophomonas, Bifidobacterium*, acetic acid, propionic acid, butyric acid, isovaleric acid, hexanoic acid, and total SCFAs were 0.6851, 0.7868, 0.8074, 0.8766, 0.7532, 0.7814, 0.8398, 0.8680, 0.8593, 0.7641, 0.7576, and 0.8658, respectively. This finding suggests that they have moderate predictive value (Swets, 1988). Investigating to the heatmap, the production of metabolites was associated with the decline in Bacteriodota, Actinobacteriota, and Firmicutes, and the increase in Proteobacteria, suggesting that it might be the joint work of the gut microbiota to produce metabolites.

This study has some limitations. Firstly, the samples were collected at a single hospital, and the cohort size was small. A large-scaled study is needed to further clarify the biomarkers in gut microbiota and SCFAs between the two groups. Secondly, as the gut microbiota composition was identified using 16S rRNA sequencing, we could not evaluate bacterial genomic functions or compare the composition at the species level. Detailed analysis using shotgun metagenomics would enable these evaluations.

## Conclusions

The composition of gut microbiota and concentrations of SCFAs of NEC infants is different from that of FPIAP infants. NEC infants had higher abundances of Actinobacteria, *Halomonas, Acinetobacter, Bifidobacterium*, and *Stenotrophomonas*, and lower abundance of Bacteroidota, lower levels of acetic acid, propionic acid, butyric acid, isovaleric acid, and total SCFAs, and higher level of hexanoic acid as compared to FPIAP infants. The differences of gut microbiota composition and concentrations of SCFAs might represent suitable biomarker targets for early identification of NEC and FPIAP.

## Data availability statement

The datasets of this study can be found in the NCBI (BioProject ID: PRJNA901061 https://www.ncbi.nlm.nih.gov/bioproject/901061).

## Ethics statement

The studies involving human participants were reviewed and approved by Institutional review board for human studies of the Children’s Hospital of Chongqing Medical University (project approval No. 2020104). Written informed consent to participate in this study was provided by the participants’ legal guardian/next of kin.

## Author contributions

All six authors made substantial contributions to the study and manuscript and met the criteria for authorship defined in the author instructions. JX drafted the manuscript, TY and X-SL collected the fecal samples and clinical data, X-CL worked on the basic sample processing, generated and analyzed the data. JX, LB, and L-QL conceived and designed the study. LB and L-QL supervised the project, contributed to the critical revision and final approval of the manuscript. All authors contributed to the article and approved the submitted version.

## Funding

The study was supported by the National Natural Science Foundation of Chongqing, China (cstc2019jcyj-msxmX0169) and Project of Chongqing Science and Technology Commission (cstc2018jscx-msybX0027).

## Acknowledgments

We thank the nurses in the Neonatal Diagnosis and Treatment Centre for collection of the fecal samples.

## Conflict of interest

The authors declare that the research was conducted in the absence of any commercial or financial relationships that could be construed as a potential conflict of interest.

## Publisher’s note

All claims expressed in this article are solely those of the authors and do not necessarily represent those of their affiliated organizations, or those of the publisher, the editors and the reviewers. Any product that may be evaluated in this article, or claim that may be made by its manufacturer, is not guaranteed or endorsed by the publisher.

## Supplementary material

Supplementary Figure 1 Rarefaction curves based on sobs index, Shannon curves based on Shannon index, and species accumulation curves between the necrotizing enterocolitis (NEC) and food protein-induced allergic protocolitis (FPIAP) groups. *N*=22 for NEC and *N*=21 for FPIAP.

## References

[B1] Abrahamsson T. R. Jakobsson H. E. Andersson A. F. Bjorksten B. Engstrand L. Jenmalm M. C. (2012). Low diversity of the gut microbiota in infants with atopic eczema. J. Allergy Clin. Immunol. 129 (2), 434–40, 440.e1-2. doi: 10.1016/j.jaci.2011.10.025 22153774

[B2] Aloisio I. Mazzola G. Corvaglia L. T. Tonti G. Faldella G. Biavati B. . (2014). Influence of intrapartum antibiotic prophylaxis against group b streptococcus on the early newborn gut composition and evaluation of the anti-streptococcus activity of bifidobacterium strains. Appl. Microbiol. Biotechnol. 98 (13), 6051–6060. doi: 10.1007/s00253-014-5712-9 24687755

[B3] Vermont Oxford Network database . Manual of operations. part 2: Data definitions and data forms for infants born in 2013. Available at: http://www.vtoxford.org/tools/ManualofOperationsPart2.pdf (Accessed 01 July 2014).

[B4] Bell E. F. Hintz S. R. Hansen N. I. Bann C. M. Wyckoff M. H. DeMauro S. B. . (2022). Mortality, in-hospital morbidity, care practices, and 2-year outcomes for extremely preterm infants in the US, 2013-2018. JAMA 327 (3), 248–263. doi: 10.1001/jama.2021.23580 35040888 PMC8767441

[B5] Berni Canani R. De Filippis F. Nocerino R. Paparo L. Di Scala C. Cosenza L. . (2018a). Gut microbiota composition and butyrate production in children affected by non-IgE-Mediated cow’s milk allergy. Sci. Rep. 8 (1), 12500. doi: 10.1038/s41598-018-30428-3 30131575 PMC6104073

[B6] Berni Canani R. De Filippis F. Nocerino R. Paparo L. Di Scala C. Cosenza L. . (2018b). Gut microbiota composition and butyrate production in children affected by non-IgE-mediated cow's milk allergy. Sci. Rep. 8 (1), 12500. doi: 10.1038/s41598-018-30428-3 30131575 PMC6104073

[B7] Biasucci G. Benenati B. Morelli L. Bessi E. Boehm G. (2008). Cesarean delivery may affect the early biodiversity of intestinal bacteria. J. Nutr. 138 (9), 1796S–1800S. doi: 10.1093/jn/138.9.1796S 18716189

[B8] Brooke J. S. (2021). Advances in the microbiology of stenotrophomonas maltophilia. Clin. Microbiol. Rev. 34 (3), e0003019. doi: 10.1128/CMR.00030-19 34043457 PMC8262804

[B9] Burks A. W. Tang M. Sicherer S. Muraro A. Eigenmann P. A. Ebisawa M. . (2012). ICON: food allergy. J. Allergy Clin. Immunol. 129 (4), 906–920. doi: 10.1016/j.jaci.2012.02.001 22365653

[B10] Calvert W. Sampat K. Jones M. Baillie C. Lamont G. Losty P. D. (2021). Necrotising enterocolitis-a 15-year outcome report from a UK specialist centre. Acta Paediatr. 110 (2), 495–502. doi: 10.1111/apa.15510 32740983

[B11] Cheffi M. Maalej A. Mahmoudi A. Hentati D. Marques A. M. Sayadi S. . (2021). Lipopeptides production by a newly halomonas venusta strain: Characterization and biotechnological properties. Bioorg. Chem. 109, 104724. doi: 10.1016/j.bioorg.2021.104724 33618256

[B12] Chen C. C. Chen K. J. Kong M. S. Chang H. J. Huang J. L. (2016). Alterations in the gut microbiotas of children with food sensitization in early life. Pediatr. Allergy Immunol. 27 (3), 254–262. doi: 10.1111/pai.12522 26663491

[B13] Curtis M. M. Hu Z. Klimko C. Narayanan S. Deberardinis R. Sperandio V. (2014). The gut commensal bacteroides thetaiotaomicron exacerbates enteric infection through modifcation of the metabolic landscape. Cell. Host. Microbe 16 (6), 759–769. doi: 10.1016/j.chom.2014.11.005 25498343 PMC4269104

[B14] de Almeida N. E. C. Esteves F. G. Dos Santos-Pinto J. R. A. Peres de Paula C. da Cunha A. F. Malavazi I. . (2020). Digestion of intact gluten proteins by bifidobacterium species: Reduction of cytotoxicity and proinflammatory responses. J. Agric. Food. Chem. 68 (15), 4485–4492. doi: 10.1021/acs.jafc.0c01421 32195585

[B15] De Filippo C. Cavalieri D. Di Paola M. Ramazzotti M. Poullet J. B. Massart S. . (2010). Impact of diet in shaping gut microbiota revealed by a comparative study in children from Europe and rural Africa. Proc. Natl. Acad. Sci. U. S. A. 107 (33), 14691–14696. doi: 10.1073/pnas.1005963107 20679230 PMC2930426

[B16] De Palma G. Cinova J. Stepankova R. Tuckova L. Sanz Y. (2010) Pivotal advance: bifidobacteria and gram-negative bacteria differentially influence immune responses in the proinflammatory milieu of celiac disease. J. Leukocyte Biol. 87 (5), 765–778. doi: 10.1189/jlb.0709471 20007908

[B17] Elizur A. Cohen M. Goldberg M. R. Rajuan N. Cohen A. Leshno M. . (2012). Cow's milk associated rectal bleeding: a population based prospective study. Pediatr. Allergy Immunol. 23 (8), 766–770. doi: 10.1111/pai.12009 23050491

[B18] Faith J. J. Guruge J. L. Charbonneau M. Subramanian S. Seedorf H. Goodman A. L. . (2013). The long-term stability of the human gut microbiota. Science 341 (6141), 1237439. doi: 10.1126/science.1237439 23828941 PMC3791589

[B19] Fallani M. Young D. Scott J. Norin E. Amarri S. Adam R. . (2010). Intestinal microbiota of 6-week-old infants across Europe: geographic influence beyond delivery mode, breast-feeding, and antibiotics. J. Pediatr. Gastroenterol. Nutr. 51 (1), 77–84. doi: 10.1097/MPG.0b013e3181d1b11e 20479681

[B20] Feuille E. Nowak-Węgrzyn A. (2015). Food protein-induced enterocolitis syndrome, allergic proctocolitis, and enteropathy. Curr. Allergy Asthma. Rep. 15 (8), 50. doi: 10.1007/s11882-015-0546-9 26174434

[B21] Geisinger E. Huo W. Hernandez-Bird J. Isberg R. R. (2019). Acinetobacter baumannii: Envelope determinants that control drug resistance, virulence, and surface variability. Annu. Rev. Microbiol. 73, 481–506. doi: 10.1146/annurev-micro-020518-115714 31206345

[B22] Hackam D. Caplan M. (2018). Necrotizing enterocolitis: Pathophysiology from a historical context. Semin. Pediatr. Surg. 27 (1), 11–18. doi: 10.1053/j.sempedsurg.2017.11.003 29275810 PMC6207945

[B23] Hua X. Goedert J. J. Pu A. Yu G. Shi J. (2016). Allergy associations with the adult fecal microbiota: analysis of the American gut project. EBioMedicine 3, 172–179. doi: 10.1016/j.ebiom.2015.11.038 26870828 PMC4739432

[B24] Ismail I. H. Boyle R. J. Licciardi P. V. Oppedisano F. Lahtinen S. Robins-Browne R. M. . (2016). Early gut colonization by bifidobacterium breve and b. catenulatum differentially modulates eczema risk in children at high risk of developing allergic disease. Pediatr. Allergy Immunol. 27 (8), 838–846. doi: 10.1111/pai.12646 27590263

[B25] Kim K. K. Lee J. S. Stevens D. A. (2013). Microbiology and epidemiology of halomonas species. Future. Microbiol. 8 (12), 1559–1573. doi: 10.2217/fmb.13.108 24266356

[B26] Kirjavainen P. V. Arvola T. Salminen S. J. Isolauri E. (2002). Aberrant composition of gut microbiota of allergic infants: a target of bifidobacterial therapy at weaning? Gut 51 (1), 51–55. doi: 10.1136/gut.51.1.51 12077091 PMC1773282

[B27] Kliegman R. M. Walsh M. C. (1987). Neonatal necrotizing enterocolitis: pathogenesis, classification, and spectrum of illness. Current. Problems. pediatrics. 17 (4), 213–288. doi: 10.1016/0045-9380(87)90031-4 PMC7130819 3556038

[B28] Korpela K. Costea P. Coelho L. P. Kandels-Lewis S. Willemsen G. Boomsma D. I. . (2018). Selective maternal seeding and environment shape the human gut microbiome. Genome. Res. 28 (4), 561–568. doi: 10.1101/gr.233940.117 29496731 PMC5880245

[B29] Korpela K. Salonen A. Hickman B. Kunz C. Sprenger N. Kukkonen K. . (2018). Fucosylated oligosaccharides in mother’s milk alleviate the effects of caesarean birth on infant gut microbiota. Sci. Rep. 8 (1), 13757. doi: 10.1038/s41598-018-32037-6 30214024 PMC6137148

[B30] Kotunia A. Pietrzak P. Guilloteau P. Zabielski R. (2010). K Butyric acid in gastrointestinal tract. Prz. Gastroenterol. 5, 117–122. doi: 10.5114/pg.2010.14137

[B31] Liang D. Zhang L. Chen H. Z. Zhang H. Hu H. H. Dai X. F. (2021). Potato resistant starch inhibits diet-induced obesity by modifying the composition of intestinal microbiota and their metabolites in obese mice. Int. J. Biol. Macromol. 180, 458–469. doi: 10.1016/j.ijbiomac.2021.02.209 33711371

[B32] Lindberg T. P. Caimano M. J. Hagadorn J. I. Bennett E. M. Maas K. Brownell E. A. . (2020). Preterm infant gut microbial patterns related to the development of necrotizing enterocolitis. J. Matern. Fetal. Neonatal. Med. 33 (3), 349–358. doi: 10.1080/14767058.2018.1490719 29909714

[B33] Lin W. Wen L. Y. Wen J. P. Xiang G. D. (2021). Effects of sleeve gastrectomy on fecal gut microbiota and short-chain fatty acid content in a rat model of polycystic ovary syndrome. Front. Endocrinol. (Lausanne). 12. doi: 10.3389/fendo.2021.747888 PMC8631770 34858330

[B34] Liu Q. Jing W. Wang W. (2018). Bifidobacterium lactis ameliorates the risk of food allergy in Chinese children by affecting relative percentage of treg and Th17 cells. Can. J. Infect. Dis. Med. Microbiol. 2018, 4561038. doi: 10.1155/2018/4561038 30651897 PMC6311867

[B35] Liu H. Wang J. He T. Becker S. Zhang G. L. Li D. F. . (2018). Butyrate: A double-edged sword for health? Adv. Nutr. 9 (1), 21–29. doi: 10.1093/advances/nmx009 29438462 PMC6333934

[B36] Lundgren S. N. Madan J. C. Emond J. A. Morrison H. G. Christensen B. C. Karagas M. R. . (2018). Maternal diet during pregnancy is related with the infant stool microbiome in a delivery mode-dependent manner. Microbiome 6 (1), 109. doi: 10.1186/s40168-018-0490-8 29973274 PMC6033232

[B37] Lu P. Sodhi C. P. Jia H. Shaffiey S. Good M. Branca M. F. . (2014). Animal models of gastrointestinal and liver diseases. Animal models of necrotizing enterocolitis: pathophysiology, translational relevance, and challenges. Am. J. Physiol. Gastrointest. Liver. Physiol. 306 (11), G917–G928. doi: 10.1152/ajpgi.00422.2013 24763555 PMC4042110

[B38] Magne F. Gotteland M. Gauthier L. Zazueta A. Pesoa S. Navarrete P. . (2020). The Firmicutes/Bacteroidetes ratio: A relevant marker of gut dysbiosis in obese patients? Nutrients 12 (5), 1474. doi: 10.3390/nu12051474 32438689 PMC7285218

[B39] Mahowald M. A. Rey F. E. Seedorf H. Turnbaugh P. J. Fulton R. S. Wollam A. . (2009). Characterizing a model human gut microbiota composed of members of its two dominant bacterial phyla. Proc. Natl. Acad. Sci. U. S. Axz. 106 (14), 5859–5864. doi: 10.1073/pnas.0901529106 PMC2660063 19321416

[B40] Markowiak-Kopeć P. Śliżewska K. (2020). The effect of probiotics on the production of short-chain fatty acids by human intestinal microbiome. Nutrients 12 (4), 1107. doi: 10.3390/nu12041107 32316181 PMC7230973

[B41] Martin V. M. Virkud Y. V. Seay H. Hickey A. Ndahayo R. Rosow R. . (2020). Prospective assessment of pediatrician-diagnosed food protein-induced allergic proctocolitis by gross or occult blood. J. Allergy Clin. Immunol. Pract. 8 (5), 1692–1699.e1. doi: 10.1016/j.jaip.2019.12.029 31917366 PMC8403015

[B42] Meyer R. Chebar Lozinsky A. Fleischer D. M. Vieira M. C. Du Toit G. Vandenplas Y. . (2020). Diagnosis and management of non-IgE gastrointestinal allergies in breastfed infants-an EAACI position paper. Allergy 75 (1), 14–32. doi: 10.1111/all.13947 31199517

[B43] Nowak-Węgrzyn A. Katz Y. Mehr S. S. Koletzko S. (2015). Non-IgE-mediated gastrointestinal food allergy. J. Allergy Clin. Immunol. 135 (5), 1114–1124. doi: 10.1016/j.jaci.2015.03.025 25956013

[B44] Ohtsuka Y. (2015). Food intolerance and mucosal inflammation. Pediatr. Int. 57 (1), 22–29. doi: 10.1111/ped.12546 25442377

[B45] Olm M. R. Bhattacharya N. Crits-Christoph A. Firek B. A. Baker R. Song. Y. S. . (2019). Necrotizing enterocolitis is preceded by increased gut bacterial replication, klebsiella, and fimbriae-encoding bacteria. Sci. Adv. 5 (12), eaax5727. doi: 10.1126/sciadv.aax5727 31844663 PMC6905865

[B46] Pammi M. Cope J. Tarr P. I. Warner B. B. Morrow A. L. Mai V. . (2017a). Intestinal dysbiosis in preterm infants preceding necrotizing enterocolitis: a systematic review and meta-analysis. Microbiome 5 (1), 31. doi: 10.1186/s40168-017-0248-8 28274256 PMC5343300

[B47] Pammi M. Cope J. Tarr P. I. Warner B. B. Morrow A. L. Mai V. . (2017b). Intestinal dysbiosis in preterm infants preceding necrotizing enterocolitis: a systematic review and meta-analysis. Microbiome 5 (1), 31. doi: 10.1186/s40168-017-0248-8 28274256 PMC5343300

[B48] Russell W. R. Hoyles L. Flint H. J. Dumas M. E. (2013). Colonic bacterial metabolites and human health. Curr. Opin. Microbiol. 16, 246–254. doi: 10.1016/j.mib.2013.07.002 23880135

[B49] Sánchez M. B. (2015). Antibiotic resistance in the opportunistic pathogen stenotrophomonas maltophilia. Front. Microbiol. 6. doi: 10.3389/fmicb.2015.00658 PMC4485184 26175724

[B50] Savage J. H. Lee-Sarwar K. A. Sordillo J. Bunyavanich S. Zhou Y. O’Connor G. . (2018). A prospective microbiome-wide association study of food sensitization and food allergy in early childhood. Allergy 73 (1), 145–152. doi: 10.1111/all.13232 28632934 PMC5921051

[B51] Schloss P. D. Westcott S. L. Ryabin T. Hall J. R. Hartmann M. Hollister E. B. . (2009). Introducing mothur: Open-source, platform-independent, community-supported software for describing and comparing microbial communities. Appl. Environ. Microbiol. 75 (23), 7537–7541. doi: 10.1128/AEM.01541-09 19801464 PMC2786419

[B52] Segata N. Izard J. Waldron L. Gevers D. Miropolsky L. Garrett W. S. . (2011). Metagenomic biomarker discovery and explanation. Genome. Biol. 12 (6), R60. doi: 10.1186/gb-2011-12-6-r60 21702898 PMC3218848

[B53] Senocak N. Ertugrul A. Ozmen S. Bostanci I. (2022). Clinical features and clinical course of food protein-induced allergic proctocolitis: 10-year experience of a tertiary medical center. J. Allergy Clin. Immunol. Pract. 10 (6), 1608–1613. doi: 10.1016/j.jaip.2022.02.013 35202870

[B54] Soldavini J. Kaunitz J. D. (2013). Pathobiology and potential therapeutic value of intestinal short-chain fatty acids in gut inflammation and obesity. Dig. Dis. Sci. 58 (10), 2756–2766. doi: 10.1007/s10620-013-2744-4 23839339 PMC4317286

[B55] Sowden M. van Weissenbruch M. M. Bulabula A. N. H. van Wyk L. Twisk J. van Niekerk E. (2022). Effect of a multi-strain probiotic on the incidence and severity of necrotizing enterocolitis and feeding intolerances in preterm neonates. Nutrients 14 (16), 3305. doi: 10.3390/nu14163305 36014810 PMC9415863

[B56] Swets J. A. (1988). Measuring the accuracy of diagnostic systems. Science 240 (4857), 1285–1293. doi: 10.1093/advances/nmx009 3287615

[B57] Tarracchini C. Milani C. Longhi G. Fontana F. Mancabelli L. Pintus R. . (2021). Unraveling the microbiome of necrotizing enterocolitis: Insights in novel microbial and metabolomic biomarkers. Microbiol. Spectr. 9 (2), e0117621. doi: 10.1128/Spectrum.01176-21 34704805 PMC8549755

[B58] Tedelind S. Westberg F. Kjerrulf M. Vidal A. (2007). Anti-inflammatory properties of the short-chain fatty acids acetate and propionate: a study with relevance to inflammatory bowel disease. World. J. Gastroenterol. 13 (20), 2826–2832. doi: 10.3748/wjg.v13.i20.2826 17569118 PMC4395634

[B59] Thänert R. Keen E. C. Dantas G. Warner B. B. Tarr P. I. (2021). Necrotizing enterocolitis and the microbiome: Current status and future directions. J. Infect. Dis. 223 (12 Suppl 2), S257–S263. doi: 10.1093/infdis/jiaa604 33330904 PMC8206796

[B60] Thompson E. C. Brown M. F. Bowen E. C. Smith L. M. vander Griten D. (1996). Causes of gastrointestinal hemorrhage in neonates and children. South. Med. J. 89 (4), 370–374. doi: 10.1097/00007611-199604000-00003 8614874

[B61] Tirone C. Pezza L. Paladini A. Tana M. Aurilia C. Lio A. . (2019). Gut and lung microbiota in preterm infants: Immunological modulation and implication in neonatal outcomes. Front. Immunol. 10, 2910. doi: 10.3389/fimmu.2019.02910 31921169 PMC6920179

[B62] Torrazza R. M. Ukhanova M. Wang X. Sharma R. Hudak M. L. Neu J. . (2013). Intestinal microbial ecology and environmental factors affecting necrotizing enterocolitis. PLoS. One 8 (12), e83304. doi: 10.1371/journal.pone.0083304 24386174 PMC3875440

[B63] Willers M. Ulas T. Völlger L. Vogl T. Heinemann A. S. Pirr S. . (2020). S100A8 and S100A9 are important for postnatal development of gut microbiota and immune system in mice and infants. Gastroenterology 159 (6), 2130–2145.e5. doi: 10.1053/j.gastro.2020.08.019 32805279

[B64] Yassour M. Vatanen T. Siljander H. Hämäläinen A. M. Härkönen T. Ryhänen S. J. (2016). Natural history of the infant gut microbiome and impact of antibiotic treatment on bacterial strain diversity and stability. Sci. Transl. Med. 8 (343), 343ra81. doi: 10.1126/scitranslmed.aad0917 PMC5032909 27306663

[B65] Ziętek M. Celewicz Z. Szczuko M. (2021). Short-chain fatty acids, maternal microbiota and metabolism in pregnancy. Nutrients 13 (4), 1244. doi: 10.3390/nu13041244 33918804 PMC8069164

