# Supplementary figures and images for: Alterations of the gut microbiota and short chain fatty acids in necrotizing enterocolitis and food protein-induced allergic protocolitis infants: A prospective cohort study
Source: Front Cell Infect Microbiol. 2022 Nov 21;12:1030588. doi: 10.3389/fcimb.2022.1030588 (PMC9720398; doi:10.3389/fcimb.2022.1030588)

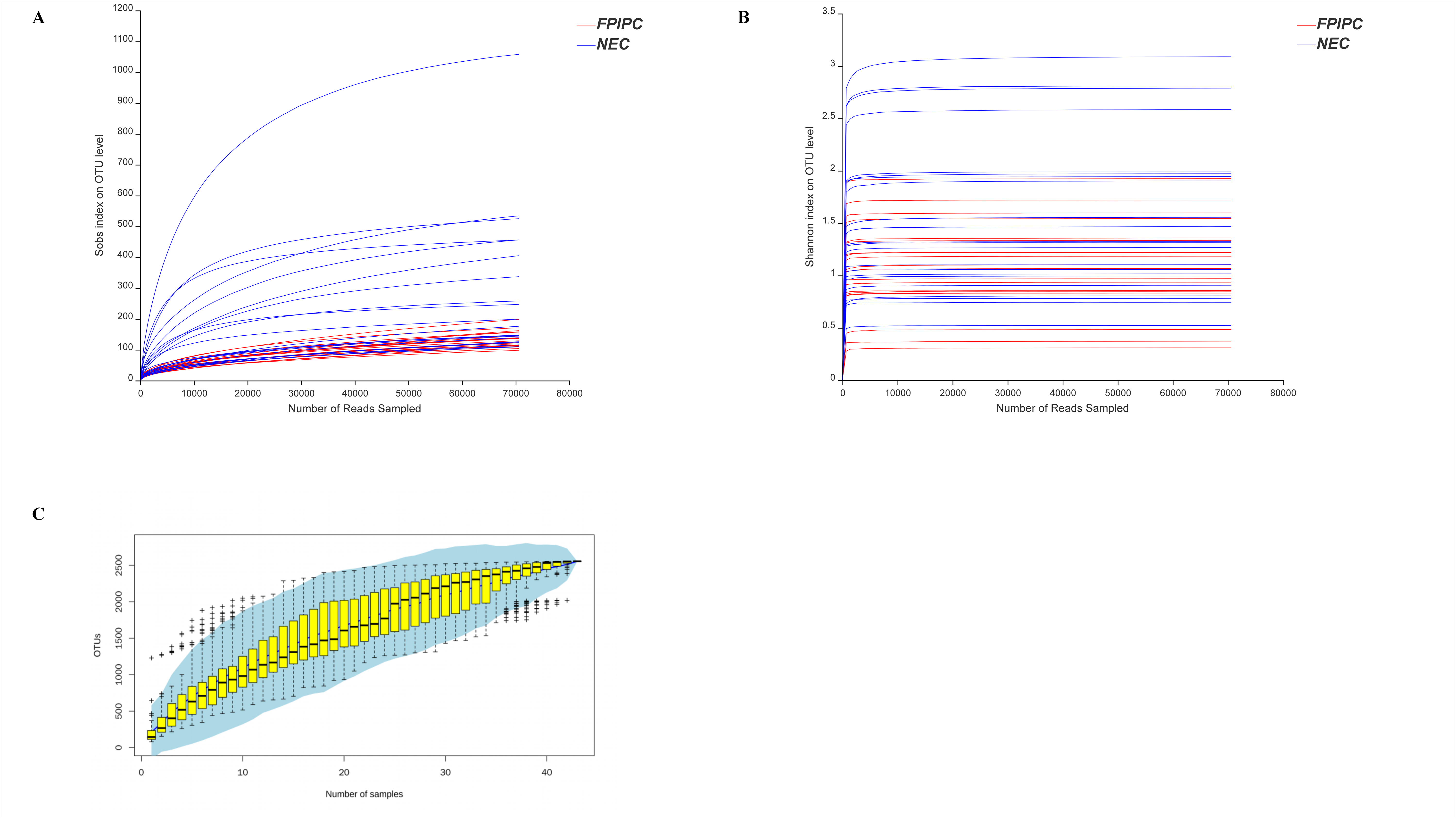

Supplement: Supplementary Figure 1 — Rarefaction curves based on sobs index, Shannon curves based on Shannon index, and species accumulation curves between the necrotizing enterocolitis (NEC) and food protein-induced allergic protocolitis (FPIAP) groups. N=22 for NEC and N=21 for FPIAP. [file Image_1.tif]
